# Two- and Three-Dimensional Tracking of *MFA2* mRNA Molecules in Mating Yeast

**DOI:** 10.3390/cells9102151

**Published:** 2020-09-23

**Authors:** Polina Geva, Konstantin Komoshvili, Stella Liberman-Aronov

**Affiliations:** 1 Department of Molecular Biology, Ariel University, Ariel 40700, Israel; polinag@bu.edu; 2 Department of Physics, Ariel University, Ariel 40700, Israel; komosh@ariel.ac.il

**Keywords:** mRNA localization, mRNA transport, mating pheromone, processing bodies, motor protein

## Abstract

Intracellular mRNA transport contributes to the spatio-temporal regulation of mRNA function and localized translation. In the budding yeast, *Saccharomyces cerevisiae*, asymmetric mRNA transport localizes ~30 specific mRNAs including those encoding polarity and secretion factors, to the bud tip. The underlying process involves RNA-binding proteins (RBPs), molecular motors, processing bodies (PBs), and the actin cytoskeleton. Recently, pheromone a-factor expression in mating yeast was discovered to depend on proper localization of its mRNA, *MFA2* mRNAs in conjunction with PBs cluster at the shmoo tip to form “mating bodies”, from which a-factor is locally expressed. The mechanism ensuring the correct targeting of mRNA to the shmoo tip is poorly understood. Here we analyzed the kinetics and trajectories of *MFA2* mRNA transport in living, alpha-factor treated yeast. Two- (2D) and three-dimensional (3D) analyses allowed us to reconstruct the granule tracks and estimate granule velocities. Tracking analysis of single *MFA2* mRNA granules, labeled using a fluorescent aptamer system, demonstrated three types movement: vibrational, oscillatory and translocational. The mRNA granule transport was complex; a granule could change its movement behavior and composition during its journey to the shmoo. Processing body assembly and the actin-based motor, Myo4p, were involved in movement of *MFA2* mRNA to the shmoo, but neither was required, indicating that multiple mechanisms for translocation were at play. Our visualization studies present a dynamic view of the localization mechanism in shmoo-bearing cells.

## 1. Introduction

Intracellular mRNA transport is one of the most important post-transcriptional mechanisms of gene regulation. The proper transport of mRNA plays an important role in localized translation, which is critical for establishing cell polarity and subcellular function. In eukaryotes, for example in fibroblasts, neurons and drosophila oocytes, it is necessary for many biological processes, including polarized growth, division, development and differentiation [1,2].

There are at least three mechanisms for localizing mRNAs in the cytoplasm: (a) directed transport to a place, (b) local protection from degradation, and (c) diffusion and entrapment by a localized anchor [3,4]. In the first mechanism, mRNAs are packaged into ribonucleoprotein (RNP) particles that are directly transported and anchored at the site of localization [5,6]. Transport of these RNP particles depends on specific *cis*-acting element(s), motor proteins and cytoskeletal filaments [7]. In the second mechanism, the RNAs are protected from degradation at one site and are highly susceptible to degradation in other parts of the cell. In the third mechanism, mRNAs freely diffuse throughout the cell but on reaching the site of localization, are unable to leave, producing a concentration “hot spot” [8].

In budding yeast, *Saccharomyces cerevisiae*, ~30 specific mRNAs localize at the bud tip, including those for polarity and secretion factors, [9,10,11]. Formation of transport-competent RNPs is initiated via the recognition of *cis*-regulatory elements present in RNA molecules by specific RNA-binding proteins (RBPs), termed trans-acting factors [4,12]. The regulatory elements are usually located within the 3′UTR [9,13]. The directed transport of *ASH1*, *SRO7,* and *IST2* mRNAs in budding yeast is mediated by RNP complexes. After transcription, maturated RNPs are exported from the nucleus, whereupon their movement through the cytoplasm to their destinations occurs on the cytoskeleton by molecular motor proteins [14] and reviewed in [3]. Localization and transport of *bicoid* mRNA in *Drosophila* and *Xdazl* mRNA in *Xenopus* oocytes as well as that of *β-actin* mRNA in fibroblast and neuronal cells have varied mechanisms principally based on RNP assembly and cytoskeletal support [4].

Understanding the dynamic behavior of mRNA movement may shed light on the mechanism by which these mRNAs localize. Methods for the dynamic study of native mRNA in living cells now exist. Current, technical developments in intracellular RNA imaging allow for observation of the movement of single mRNA molecules in living cells in real time [7,15,16]. One popular approach is to insert a linker site for RBP within the 3′UTR of the mRNA of interest. The RBP is conjugated to a fluorophore that enables microscopic tracking of individual mRNA transcripts in living cells [7,17]. To track single mRNA movement in real time, it is important to achieve high sensitivity for single molecule detection and fast image acquisition. A sufficient tracking range is crucial to identify the type of motion. The total number of frames in the image sequence determines the statistical accuracy of the analysis.

Most studies were performed in 2D, but it is highly desirable to extend the technology into 3D because most biological processes occur within a volume [18]. Recently, single mRNA particles in live budding yeasts were tracked in three dimensions using a microscope with a double-helix point spread function [6,19]. Simultaneous observation of *ASH1* and *IST2* mRNAs revealed directed co-transport from the mother cell into the bud, followed by corralled movements of both mRNAs once they reached the bud cortex [19].

In the cytoplasm, mRNAs are always chaperoned by RBPs that create RNP complexes [20]. These complexes can be quite variable in size, properties, and function, which is dependent on its contents. Processing bodies (PBs) are a special type cytoplasmic RNP complex, primarily composed of translationally repressed mRNAs and proteins related to mRNA decay, suggesting roles in post-transcriptional regulation [21,22]. PBs may also play a role in mRNA transport as evidenced by the various types of PB movements in different cell types. [23,24,25]. The mRNAs transported within PBs to specific locations are protected from degradation and preliminary translation by RBPs present in the complex which are also regulated in their transport to specific destinations [25,26].

In budding yeast, mRNPs transport is associated with multiple Myo4p motors via the activity of the RBPs, She2p and adaptor She3p [3,27]. Assembled RNPs are transported from the mother into the bud tip by actomyosin-based transport [28,29]. Transported mRNPs accompany cortical endoplasmic reticulum (ER) using the SHE family proteins [30,31]. RNP granules and ER movement is consistent with the speed generated by a motor. The *ASH1* mRNA is contained in RNPs that move at velocities that vary between 0.2–0.44 µm/s [7], which are consistent with a myosin V motor [32]. Cell-specific RNA velocity estimates provide a natural basis for intracellular mRNA behavior during cell cycle. The PBs containing mRNA/RBPs complexes promote not only transport from mother to daughter cell, but also mRNA local translation required for gene function [27,33].

Recently, attention has turned to mRNA transport in mating yeast. The advantage of this system over budding yeast is that in order to mate, haploid cells of the opposite genotype (*MATα/a*) create an asymmetrically growing long protrusion called a shmoo in response to pheromone treatment. It allows for the investigation of mRNA transport behavior and its trajectory over long distances, similar to axonal transport in neuronal cells [34,35,36]. Our previous research uncovered the role of mRNA transport and localization in the regulation of aF translation in mating yeast. We demonstrated that in response to pheromone treatment, cytoplasmic *MFA2* mRNA is transported to the shmoo tip where it is locally translated [27]. Only a small fraction of the mRNA granules located in mother cells shows direct rapid movement from the cell body to the shmoo tip where it accumulates as one large granule named “mating body.” These transported mRNPs are associated with the PB marker, Dcp2. *MFA2* mRNA is eventually released for local translation of a-factor (aF). aF is exported out of the cells by the ATP binding cassette (ABC) transporter Ste6p at the shmoo tip for activation of effective mating with a potential partner [33]. For comparison, an unrelated mRNA, *PGK1* (Phosphoglycerate Kinase 1) that is translated to a glycolytic enzyme, distributes randomly throughout the cell. No directed *PGK1* mRNA transport to the shmoo was found using two-dimensional video analysis [27]. The mechanisms responsible for correctly targeting proteins to the shmoo tip are not well understood.

The *MFA2* mRNA showed distinct subgroups of the granules, which were different in location in the cell, size, and motion properties. In our previous studies, we carried out two-dimensional imaging studies (2D); here, we investigated the dynamics of all types of *MFA2* mRNA granules with a high spatial resolution during shmoo growth using four dimensional studies (4D), including z- stack and time intervals, which were transformed to 3D for analysis that should give more accurate tracking information about position, behavior and velocity of the granules than 2D. We addressed several questions: What trajectories do they exhibit? Three-dimensional measurements are essential to extract full information about mRNA dynamics during movement including physical properties. What factors participate in their movement? What role does a motor Myo4p play in *MFA2* mRNA delivery to the shmoo? To address these questions, we described and classified mRNA movements in *S. cerevisiae* after stimulating *MATa* cells expressing fluorescently labeled *MFA2* mRNA with α-factor mating pheromone.

## 2. Results

We investigated the dynamics of *MFA2* mRNA granules labeled with U1A-GFP in α-factor treated, WT yeast cells (ySA056). In parallel, we examined the behavior of *PGK1* mRNA that expressed transcript from a housekeeping gene, *PGK1,* (ySA061) as a control unrelated to mating. We used a previously developed system consisting of two plasmids encoding *MFA2-U1A* binding sites and U1A-GFP [17,37]. To allow for live-cell imaging of individual mRNA granules, each transcript insert contained 16 repeated aptamer fragments in its 3′UTR region to enable recognition by U1A RNA-binding protein fused with GFP. The aptamer-tagged mRNAs were localized using fluorescence microscopy. To validate the specificity of targeting of the transcript to shmoo and the possible influence of the labeling method, we generated a plasmid that contained U1A-loops only and it was co-expressed with the plasmid for U1A-GFP expression. The strain (ySA180) expressing transcript with U1 loops only showed 33% background localization in the shmoo tip vs. 73% in the strain expressing the full version of the *MFA*2 mRNA including the loops (ySA056, Figure S1, panel A). Therefore, we concluded that *MFA2* mRNA delivery to shmoo was U1A loops independent. This labeling system was also used in identifying 22 bud-specific localized transcripts present in complex with She family proteins and delivered to the bud in an actin-myosin fashion for their bud segregated protein activity [38]. Today it is one of the most efficient labeling systems for studying mRNA transport in living cells. However, we cannot exclude that changes in length of *MFA2* gene and creation of constructs longer than the physiological one can be affected by transport behavior of the transcript.

All cells that expressed *MFA2* mRNPs under observation formed only a single shmoo, suggesting that all responsive cells possessed unidirectional polarity. Randomly chosen cells with different stages of shmoo development were used for imaging. Within a given cell, individual granules moved independently and in different directions, indicating that their motion was not due to bulk movement of the cytoplasm.

### 2.1. Range of Movement of MFA2 and PGK1 mRNA in 3D

**Figure 1:**
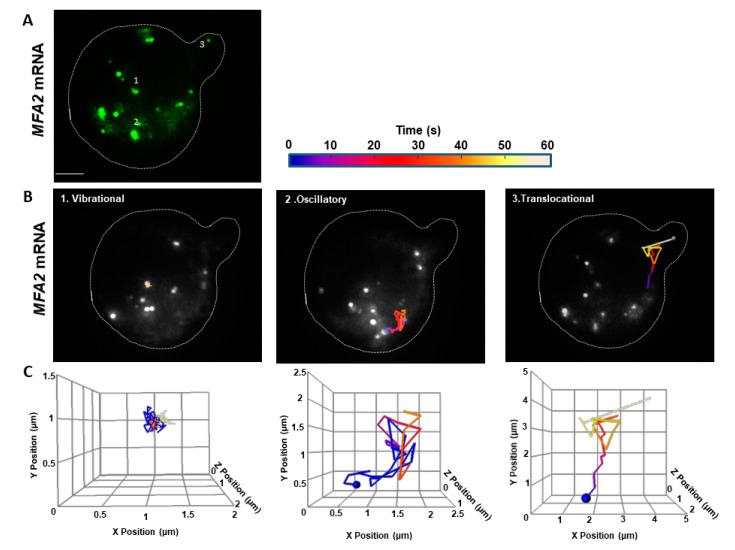
*MFA2* granules exhibited three types of motion in 3D tracking. (**A**) U1A-GFP labeled *MFA2* mRNA in a yeast cell were visualized after treatment with α-factor for 2 h. Broken line outlines the cell border with a shmoo developing on the upper right side. Numbers refer to particles tracked in (**B**,**C**). Scale bar = 2 µm also applies to (**B**). (**B**) Tracks of three particles display distinct behavior movements: vibrational, oscillatory, and translocational were superimposed on images of the cell at various fixed times. Tracks were constructed from images recorded every 4 s, consolidating 6 z-stacks with a depth of 0.5 µm for each cell. (**C**) Tracks of the *MFA2* granules shown in (**B**) are shown replotted in 3D. The axes were arbitrarily resized to illustrate the movement space of the granules. Blue spheres mark the initial positions of the granules. Track duration was 60 s.

To understand the mechanism of localizing *MFA2* granules to the shmoo tip, we compared the movement of *MFA2* and *PGK1* granules in 3D. Similar to what we have published previously in 2D [27], there were three essential types of *MFA2* granule motion in 3D by using Image Analysis Software (Imaris) (Figure 1, Methods, Movie S1): vibrational, oscillatory and translocational that have distinct sizes, locations in the cells, and behavior. Only two types of *MFA2* mRNA granules were motile: oscillatory and translocational. The vibrational and oscillatory *MFA2* mRNA granules were mostly observed in the main cell body, whereas translocated mRNA granules were more motile and were delivered to shmoo. Figure 1A shows a representative image of several *MFA2* mRNA containing granules that vary in size from 0.8 to 0.2 μm. Size was dependent on the number of copies of mRNA molecules per granule and/or RBP types associated with it.

A “track displacement” parameter, defined as the change in position of an mRNA granule relative to its starting point, was used to classify movement type of *MFA2* RNP (Table 1). For normalization, this parameter and the overall distance traveled (track length), were calculated for 60s periods (see Methods). For *MFA2* mRNA granules undergoing translocational movement, track displacements ranged from 1.5 to 4.0 μm, with a mean of 2.3 μm. Translocational movement appeared to be restricted to low intensity granules, 0.2 to 0.5 μm in diameter. The lowest intensity mRNA granules (0.2 μm) were actively transported to shmoo tips (Figure 1B,C) and they contained up to 10 mRNA transcripts, according to previous estimates [39]. Oscillating granules traveled the same overall distance (track length) in the same period as translocating granules, but their track displacement was twofold less (1.1 μm). Their movement was restricted to a local area inside of the cell body and they only moved a slight distance away from the starting point, as compared to the translocational granules. For granules undergoing vibrational movement, track displacement was limited to 0.8 µm and was fourfold less as compared to translocated granules. They appeared to be attached to a place and displayed characteristics of immotile granules. Granules undergoing oscillatory or vibrational movement ranged widely in size; our analysis focused on those varying from 0.2 to 0.8 μm in diameter.

**Figure 2:**
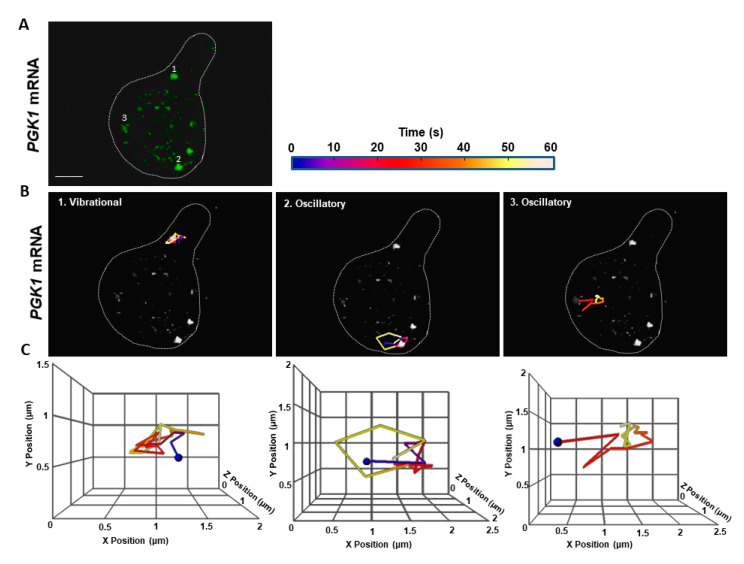
Only vibrational and oscillatory motions occurred in *PGK1* granules in 3D. The explanation is similar to that for Figure 1, except that this cell expressed U1A-GFP labeled *PGK1* mRNA. *PGK1* granules did not exhibit translocational movement. Scale bar = 2 µm.

Unlike the *MFA2* granules that moved a significant distance from the cell body to the shmoo (Figure 1B,C, Table 1), *PGK1* granules were smaller, ranging in size from 0.1–0.5 μm. They were more motile, although they moved only within limited sub-regions and were never observed to translocate to other cell compartments over periods of 60 s (Figure 2, Movie S2). However, track lengths for oscillatory *PGK1* granules were 2.3-fold greater than for *MFA2* granules (*p* < 0.001), due to the more frequent occurrence of local jumps (see following sections). Vibrational track displacements of *MFA2* and *PGK1* granules were similar, but track length may have been several times larger for the latter (Table 1). The distinct behavior of *PGK1* mRNA granules could be due to their with a different granule composition, which is known to be important for regulation of granule stability, translational rate, and degradation.

To summarize, according to 3D analysis, most *PGK1* mRNA granules showed strong, local motile behavior, while none translocated to the shmoo. *MFA2* mRNA granules also showed restricted to local dynamic movement albeit to a somewhat lesser extent, but in addition, some low intensity *MFA2* granules underwent directed movement toward the shmoo.

### 2.2. Comparison of MFA2 Granule Movements in 2D and in 3D

**Figure 3:**
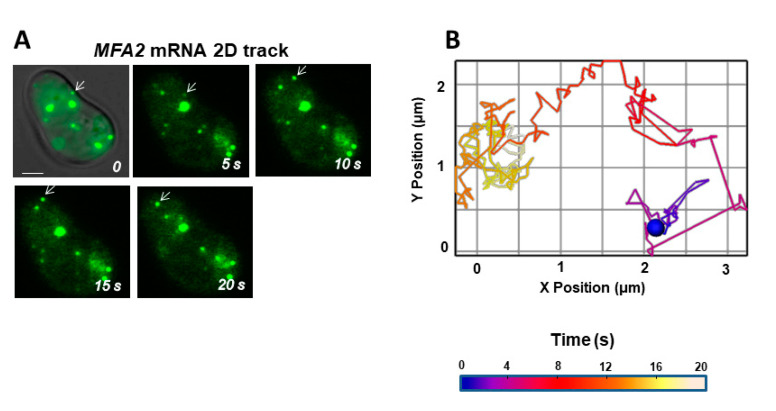
High time resolution tracking in 2D revealed extensive *MFA2* granule movement. (**A**) Cells expressing mRNA (green) treated with α-factor for 2 h were examined by fluorescence microscopy. There was a rudimentary shmoo on the upper left side of the cell. White arrow indicates the motile, low intensity mRNA transport granule analyzed in (**B**). Scale bar = 2 µm. (**B**) Over most of its journey, the granule changed directions incessantly and took a very indirect route to the shmoo. The color scale refers to the time. Track displacement rate was 0.01 μm/s. Mean velocity was 0.73 μm/s.

To find out whether a significant amount of movement occurred on a fast time scale, mean velocity and track displacement rate of the fastest subset of translocating *MFA2* granules (0.2–0.4 μm in diameter) were examined in 2D using a very sensitive camera mounted on a standard fluorescent microscope that allowed volume shooting of the granules without z stack scanning per frame (see Methods), (Figure 3, Movie S3). Sacrificing information about granule movement in the *z*-axis reduced frame capture time to 0.055–0.070 s. For translocating granules, the mean velocity calculated from the total track length in 2D and the track duration was 1.5 µm/s. In contrast, “track displacement rate” determined as 2D track displacement divided by track duration was only 0.04 μm/s (Table 2). The discrepancy between mean velocity and track displacement rate reflected the circuitous route traveled as well as several periods of aimless wandering.

**Figure 4:**
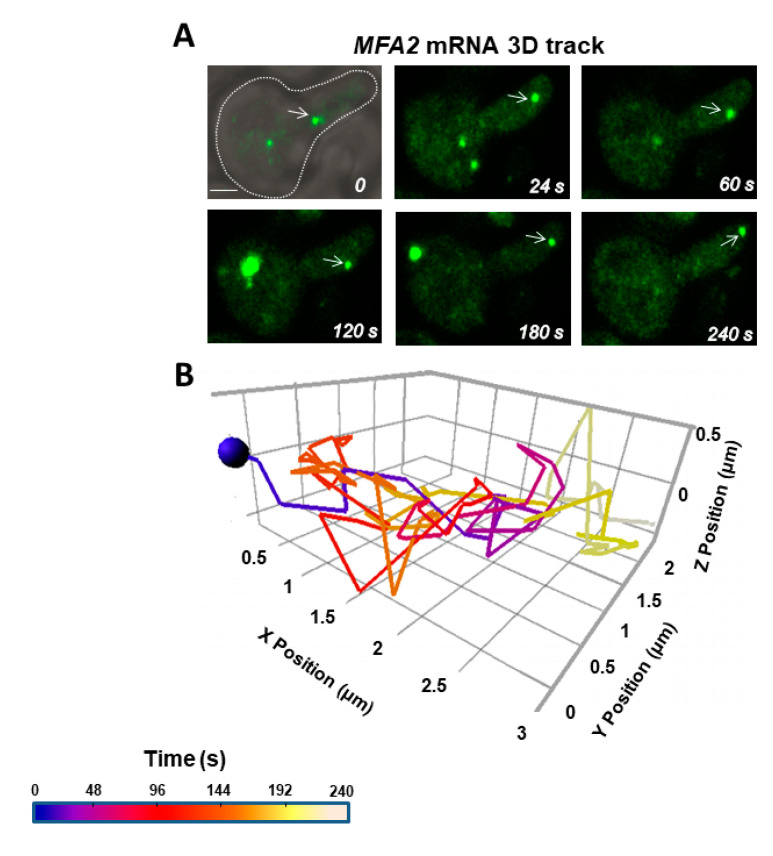
A translocating *MFA2* granule made numerous twists and turns in the z- direction. (**A**) Cells expressing labeled *MFA2* mRNA (green) and treated with α-factor for 2 h were recorded in 3D by confocal microscopy. White arrows indicate the motile, low intensity, transport mRNA granule analyzed in (**B**). Scale bar = 2 µm. (**B***)* Although route mapping in 3D was coarse grain compared to that in 2D, frequent detours in the z-direction were apparent. The color scale refers to the time. Track displacement rate was 0.01 μm/s, mean velocity was 0.09 μm/s.

Motile, low intensity mRNA granules 0.2–0.5 μm in diameter were analyzed for 3D mRNA transport in real time using confocal microscopy (Movie S4). The cells typically had a thickness of 3-4 μm. These granules were similar to the granules analyzed in 2D but included some that were somewhat larger and/or slightly more intense. The lowest intensity, fastest-moving *MFA2* granules that also had directed movement as shown previously in 2D here and in [27] were not accessible for the 3D analyses. Z scanning increased significantly the frame acquisition time to 2–4 s and we were able to follow more accurately, the details of the RNP tracks as compared to 2D. Figure 4A and Movie S4 show an example of 3D tracking at 2 s/frame, of an mRNA granule as it moved from the neck to the tip of the shmoo. The average velocity for 10 granules was 0.15 μm/s and the average of the track displacement rate was 0.03 μm/s (Table 2). Compared to the analysis in 2D, the speed estimated in 3D was 10-fold slower, while the track displacement rate was similar. To rule out a systematic bias in granule selection by size and fluorescence intensity in 2D versus 3D imaging, the 2D velocities of four translocational granules were recalculated for 2 s intervals, yielding a range of 0.08–0.2 µm/s that corresponded more closely to values obtained with 3D analysis. Thus, the discrepancy in mean velocity reflected a limitation in the rate of frame capture, which was 60 to 80 times faster in 2D than in 3D imaging.

In both cases (2D and 3D), the translocational movement did not take the shortest route from the mother to the shmoo tip. The granule typically went through many twisting and rotary motions close to its initial location before performing one or more large directed jumps toward the shmoo (Movies S1, S3, and S4). However, it became clear that “straight” segments in 2D usually involved deviations in the z-direction.

Interestingly, granules arriving at the shmoo tip did not lose the ability to move; they instead reverted to local oscillation similar to what was typical prior to a jump (e.g., Figure 3). Normally, pheromone binding to G-protein coupled receptors stimulates polarized growth in one direction, toward the source of the pheromone gradient established by the nearest cell of opposite haplotype [40]. Granules did not stop moving at their destination in this experimental setting, possibly because the α-factor was present in the medium all around the cell. In addition, directed granule movement may have had to wait for further maturation of the shmoo, since shmoo extension was incomplete in many of the cells under study.

### 2.3. Physical Movement Parameters of MFA2 and PGK1 Granules

**Figure 5:**
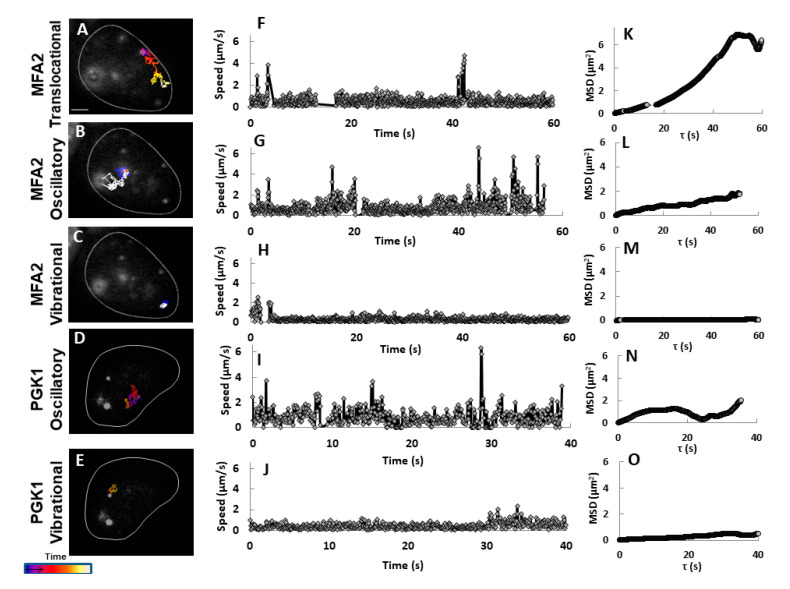
MSD rather that velocity distinguished the different types of movement. Three *MFA2* (**A**–**C**) and 2 *PGK1* (**D**–**E**) granules were tracked in yeast cells treated with pheromone for 2 h before movies were taken in 2D by fluorescence microscopy. The interval between frames was 0.055–0.07 s. Scale bar = 1.5 µm applies to (**A**–**E**). (**F**–**J**) Speed at a given time point was given as the average of the local velocities determined for three neighboring time points, where local velocity was the calculated as the distance traveled over two frames divided by one time intervals. (**K**–**O**) Mean Square Displacement (MSD) was diagnostic for random and nonrandom translocation as well as corralled movement.

Next, we tested the randomness of granule movement by examining the nature of motion on a fine time scale, supported by known physical parameters. Two-dimensional analysis was more appropriate, because greater time resolution was required. Fluorescent labeled *MFA2* and *PGK1* mRNAs were imaged in yeast that were treated with α-factor for 2 h to induce shmoo growth. The cells were recorded at 14–18 frames/s for 60 s. Figure 5A–E shows representative *MFA2* and *PGK1* granule tracks of the various movement types.

Two additional parameters were analyzed: local velocity and mean square displacement (MSD). Local velocity at a given time point was estimated from the 2D position immediately preceding and the position immediately following that time point. Both vibrating *MFA2* and *PGK1* granules showed start-stop movement and relatively slow local velocities when moving, with a few bursts up to 2 μm/s (Figure 5H,J). Interestingly, there were high intensity *MFA2* mRNPs (size 0.5 µm) containing 40–50 mRNA copies, located in the shmoo (Figure 5C,H,M). These granules were Mating Bodies that play a role in controlling the translation of aF. Based on MSD analysis the Mating Bodies were attached within the shmoo tip. Oscillatory RNPs granules of both mRNA types had similar local velocities fluctuating between 0 and 2 μm/s that were punctuated by high speed jumps of up to 5–6 μm/s (Figure 5G,I). We found that small *PGK1* granules did not translocate to the shmoo, but *MFA2* granules undergoing translocation behaved like oscillating granules in terms of local velocity (Figure 5F), although they sometimes reached speeds as high as 8 μm/s. However, the MSD profiles associated with these two types of movement were completely different.

The MSD represents an ensemble average in the sense that it measures the spreading of many particles, characterized by the spatial average of squared particle position [41], (see Methods). According to Brownian motion theory, MSD is proportional to the time during which the movement is random. If movement is directed, then the MSD function tends to follow a parabolic or an exponential function over time. When the MSD profile decreases it is a “corralled” motion due to some local potential [42]. Figure 5K shows that while an *MFA2* granule was undergoing translocation (Figure 5A), the MSD function was parabolic. In contrast, one oscillatory granule (Figure 5B) showed a linear MSD graph (Figure 5L) that indicated random movement. Hence, the MSD parameter demonstrated that despite the similar distributions of local velocities between the two types of granules, the nature the movement was quite different: in Figure 5A it was directed transport until it reached the shmoo and in Figure 5B, it was random walk. The *PGK1* mRNA granule in Figure 5D exhibited oscillatory movement within a confined area hence the MSD profile remained below a maximal value and included a segment with a negative slope. Vibrational *MFA2* and *PGK1* granules (Figure 5C,E) did not change their locations by more than 0.9 μm and were marked by MSD profiles with very low slopes (Figure 5M,O). Thus, vibrational granules were tethered or corralled and probably not engaged in very slow, directed movement.

### 2.4. Variable Movement of MFA2 Granules within a Single Track

**Figure 6:**
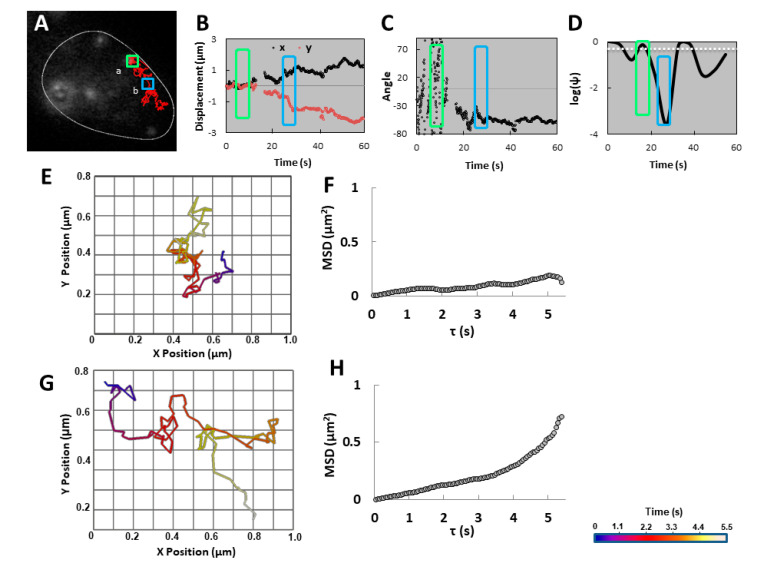
Transitions in type of movement were observed in the same track. (**A**) Two sections of the track for the granule in Figure 5A, marked by the green and blue squares, were analyzed further in 2D in panels (**B**–**F**). (**B**) Granule displacements on the x- and y- axes as a function of time were minimal at first (green box), but then increased in magnitude over time (blue box). (**C**) Angle of the movement relative to its position at the start of the observation period as a function of time was initially random (green box), then turned several times (e.g., blue box), before settling on a particular directionality. (**D**) Log(ψ) of the track was calculated in segments of 100 frames as a function of the time. The white dashed line represents the Log(ψ) threshold = −0.3 Log(ψ) falling below the threshold was indicative of directed motion. (**E**,**G**) Vantage visualizations are shown for the track segments marked by the green and blue boxes in (**A**), respectively. The color scale refers to different time periods lasting 5.5 s in these two panels only. (**F**,**H**) MSD parameter was calculated for the track segments marked by the green and blue boxes in (**A**), respectively.

*MFA2* granules undergoing translocation often appeared to engage in a different type of movement initially (e.g., Figure 3 and Figure 5A). To explore these granules further, we examined their changes in position relative to the starting point in the x- and y-directions separately over time, the angle of movement, and a ψ parameter that indicated the probability that a granule would stay within a circle of maximum displacement. (See Methods, Appendix S1). Figure 6 shows the analysis of the *MFA2* granule in Figure 5A as it moved from the cell body to the shmoo, focusing on two sections of the track. The first consisted of 100 frames in the initial, 5.5 s segment of the track (green square in Figure 6A) in which the granule oscillated within a limited region. The second section (blue square in Figure 6A) includes 100 frames in which the *MFA2* mRNA granule translocated. These two tracks, made by the same granule, differed substantially with regard to all of the parameters analyzed. Possibly, this RNP was subjected to a switch in regulation due to an exchange of its RBP components.

The green section was marked by a lack of order in terms of positional change (Figure 6B) and angle of movement (Figure 6C). Vantage visualization of the granule route revealed a track displacement of only 0.21 µm (Figure 6E) and a linear MSD profile (Figure 6F). Furthermore, the parameter log(ψ) exceeded the threshold (see Methods), suggesting a high probability of the granule remaining within a region of maximum displacement of 0.11 µm, in this track fragment (see Methods) (Figure 6D). Hence, this small, low intensity *MFA2* granule initially seemed to move randomly without venturing far from its starting point.

In contrast, in the blue section there was a fairly steady increase in displacement away from the starting point in both x- and y-axes (Figure 6B) and a constant rate of change in the angle of movement (Figure 6C) overall. Finer time resolution revealed greater complexity with some backward jumps along one axis and a few very brief epochs that mimicked vibrational movement (Figure 6G). The log(ψ) parameter was much lower than in the green section predicting a lower probability of the granule staying within a circle of maximum displacement of 0.14 µm, in this track fragment (Figure 6D). The highlight was the MSD plot for this section of movement with a parabolic profile indicating directed rather than random motion. It is noteworthy that even when analyzed over the entire 60 s period during which movement was nonuniform and riddled with local wandering (producing a variable log(ψ), Figure 6D), jitters and jumps with velocities up to 4 µm/s (Figure 5F), the MSD profile was still parabolic for most of the track (Figure 5K).

These findings belie movement that was much more complex than was apparent from lower time resolution imaging. Different factors regulate the mRNA movement in different parts of the cell and probably there were numerous underlying mechanisms.

### 2.5. MFA2 Granules Co-Transported with PB Proteins to the Shmoo Tip

**Figure 7:**
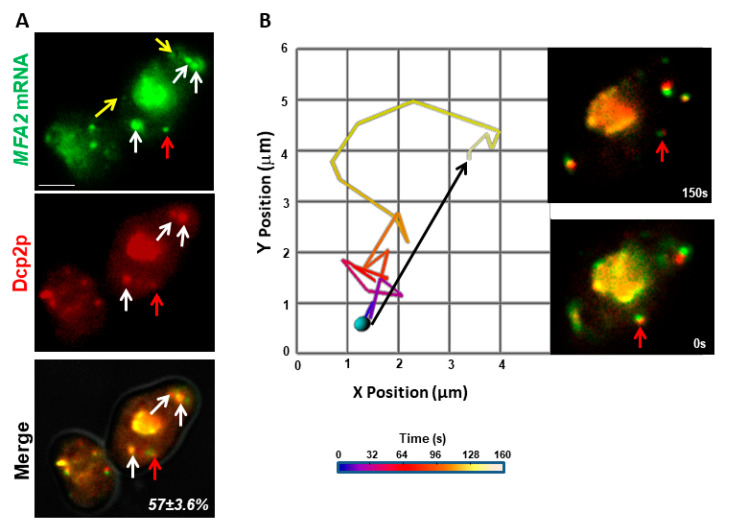
*MFA2* mRNA and the PB marker co-transported to the shmoo tip. (**A**) *MFA2* mRNA (green) co-localized with Dcp2p-RFP (red), a PB marker, in many granules. Several small co-labeled granules are indicated by the red and white arrows, while granules with *MFA2* alone are marked by yellow arrows. Scale bar = 5 µm. (**B**) The 2D track is shown for the granule in (**A**) indicated by the red arrow. The track displacement of this translocational granule (black arrow) over an observation period spanning 150 s, was 3.8 µm. Inset: the two markers remained co-localized during the entire track.

Since *MFA2* mRNA co-localizes with PB proteins in the shmoo tip in α-factor treated cells [27], the question arose as to whether the mRNA trafficked as PBs, or whether they formed PBs once they arrived at the destination. To find out, the *MFA2* mRNA labeling system was used along with an additional plasmid for Dcp2p-RFP, a PB component coupled to a different fluorophore. In three independent experiments, 85 granules in 16 cells analyzed separately for movement of *MFA2* mRNA (GFP fluorescence) and PBs (RFP fluorescence). A cell with seven granules is shown in Figure 7 and in Movie S6. The bulk of granules in the center and four separate granules (three white arrows and one red arrow) co-labeled for PB protein and *MFA2* mRNA. Co-labeled granules displayed all three types of motility and the mean track velocities determined for each granule by following GFP and RFP separately, showed good correspondence: vibrational (0.05 µm/s), oscillatory (0.15 µm/s) and translocational (0.13 µm/s), indicating that PB proteins accompanied *MFA2* mRNA in the transport, localization, storage and sorting processes (Table 3). Figure 7B shows the route of one *MFA2* mRNA-PB as it translocated from cell body to shmoo. Moreover, this track was very reminiscent of the translocational tracks examined in Figure 1 and Figure 3, after taking into account the different rates of frame capture.

Consistent with our previous research [27], all high intensity granules were co-localized with Dcp2p-RFP, whereas 53% of low intensity granules contained this protein. A total of 57% of the *MFA2* granules were co-labeled by the PB marker (Figure 7A, yellow arrows). Because Dcp2p is a core PB component [43], we considered the rest of the low intensity granules that did not co-localize with Dcp2p, not to be PBs. However, quite possibly, the amount of Dcp2p in these granules was below microscopic sensitivity and therefore was not observable. *MFA2* mRNA granules lacking Dcp2p showed comparable oscillatory movement but a translocational movement with a two-fold faster mean velocity (Table 3), compared to granules of the same size that bound Dcp2p. Interestingly, vibrating mRNA granules missing Dcp2 were not observed. Granules with Dcp2p alone were not observed indicating that all PBs contained *MFA2* mRNA. Therefore, we can conclude that translocation of *MFA2* granules could occur without PB formation, but that PBs participated in at least a fraction of *MFA2* mRNA transport to the shmoo tip (See Discussion). It follows that PB formation affected the modes of movement available to a granule.

### 2.6. MFA2 mRNA Localization in the Shmoo Tip Was Dependent on the Actin Cytoskeleton

**Figure 8:**
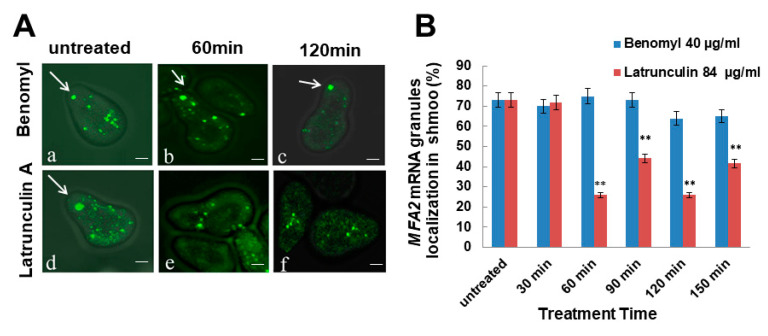
*MFA2* mRNA containing granules dissociated in response to Latrunculin, but not Benomyl treatment. (**A**) Cells expressing *MFA2* mRNA were treated with αF for 2 h. Benomyl (40 μg/mL) or lantunculin (84 μg/mL) was added to the cells which were then observed by fluorescence microscopy for 150 min. White arrows indicate *MFA2* mRNA localization in the shmoo tip. (**B**) The percentages of high intensity granules in the shmoo were calculated before and up to 150 min after addition of the drug. Error bars represent the standard deviation from three independent experiments. The star signifies a significant difference (*p* < 0.001) of *MFA2* mRNA distribution compared to untreated controls. 200–250 cells were counted. Scale bar = 2 µm.

The transport and localization of mRNAs to the daughter cell in dividing yeast occurs via the actin cytoskeleton and requires motor Myo4/She1 [9,10]. Transport of mRNAs in mating yeast was examined in the cellular responses to pheromone only for a few genes. The *SRO7* and *FUS3* mRNAs were localized to the shmoo tip on alpha mating factor treatment [14]. We have recently shown that the *MFA2* mRNA was transported and localized in the shmoo tip in αF pheromone-treated cells, but the question of how mRNA was delivered to the shmoo tip remained unexamined. To find out if actin cables or microtubules (MTs) were required for delivery, cells expressing fluorescent labeled *MFA2* mRNA (ySA056) were induced by αF for 2 h, and the cells that formed a shmoo were then treated for 2.5 h with benomyl to disrupt MTs or with latrunculin A to depolymerize actin. Fluorescence microscopy was used to examine mRNA localization in the cells after treatment for 30, 60, 90, 120 and 150 min (Figure 8A).

75% of *MFA2* mRNA granules were transported and localized to the shmoo following incubation with αF for 2 h in untreated cells (Figure 8). In cells treated with the actin-disrupting agent lantrunculin A, mRNA localization decreased to 30% after 60 min and remained low during the experiment’s duration, 150 min. The number of highly motile granules dramatically decreased by 60% (Figure 8B). Lantrunculin A is known to bind actin monomers and hinder polymerization of actin filaments. Therefore, actin filaments existing in the cell continued to function for a certain time (up to an hour), but new actin filaments could not form and therefore newly synthesized *MFA2* mRNA granules could not reach the shmoo. Consequently, after 30 min the percentage of localized mRNA was unaffected by Lantrunculin A treatment, while after 60 min, their number had decreased (Figure 8B).

Such effects were not observed in Benomyl-treated cells, where transport and localization of mRNA was similar to that observed for control cells. These results indicate that it is the actin cytoskeleton, and not MTs that plays a role in delivering *MFA2* mRNA to the shmoo (Figure 8B).

### 2.7. Myo4p Involvement in MFA2 mRNA Transport

**Figure 9:**
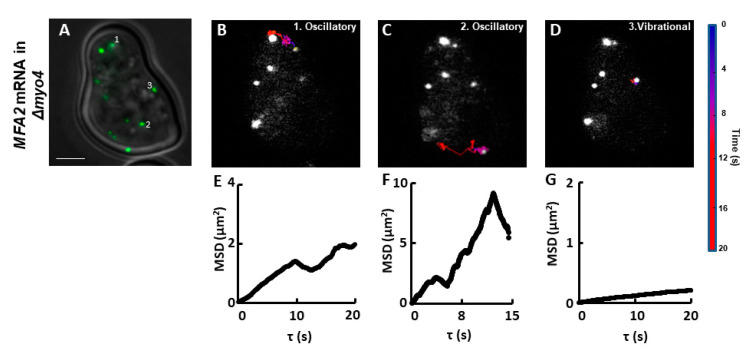
*MFA2* granules in *Δmyo4* yeast did not exhibit fast translocational movement to the shmoo. (**A**). *ΔMyo4* cells expressing labeled *MFA2* mRNA were treated with pheromone for 2 h and recorded at 0.055–0.07 s time intervals for 60 s. Scale bar = 2 µm also applies to (**B**–**D**). (**B**–**D**) Tracks of three particles in mutant cells were limited to two motility types: vibrational and oscillatory. The color scale refers to time. (**E**–**G**) Each MSD profile corresponds to the track presented above it in panels (**B**–**D**).

Deletion of Myo4p, the main motor protein in budding yeast, did not prevent *MFA2* mRNA localization or inherence of cortical ER to the shmoo (Figure S1 panel B). Here we investigated whether the absence of Myo4p changed the movement of *MFA2* granules in a discernible way. *MFA2* mRNA was labeled in a yeast strain lacking Myo4p (*Δmyo4)*. After cells were treated with α-factor for 2 h to induce shmoo formation, 2D tracking analysis was performed on images recorded at 14–18 frames/s for 60 s. Although the localization percentage of *MFA2* granules at the shmoo was similar to that in WT yeast, granules translocating from cell body to shmoo were never observed in mutant cells (Figure 9 and Movie S7). All movements were either vibrational (Figure 9D,G), oscillatory in which the granules moved in a confined area (Figure 9B,E), or oscillatory with larger displacements not directed toward the shmoo (Figure 9C,F). According to the displacement parameter the latter can be considered to be translocational, but the overall track MSD profile was linear (See also Discussion). The movement types were verified by the analysis of MSD. The diffusion coefficient was less than or equal to 1 µm^2^/s. Similar to WT, “corralled motion” indicated by a negative slope of the MSD profile was observed (Figure 9C,F). Thus, while *MFA2* mRNA localization did not strictly require the presence of Myo4 motor protein, the mRNA movement was affected. It implied that one mode of transport to the shmoo was blocked by Myo4p deletion, so the *MFA2* mRNA that did localize to the shmoo: translocated as very small granules not detected by our methods, were possibly carried by a different motor protein, or used another mechanism entirely.

Table 4 compares the granule velocities of the WT strain versus the mutant *Δmyo4* strain. Granules undergoing vibrational and oscillatory movements showed very similar velocities between strains. This finding emphasizes that these two types of granule movements were unaffected by the absence of the Myo4p motor, and only the ability to move in a directed fashion to the shmoo was lacking. Yet somehow, *MFA2* granules still localized in the shmoo.

## 3. Discussion

Here, for the first time, we examined *MFA2* and *PGK1* RNP transport in high resolution and characterized their motile properties in αF treated cells.

### 3.1. Comparison of 2D and 3D Tracking

Research into the mRNA dynamics in yeast has been conducted mainly in two dimensions. Notably, there are two 3D studies: the first simultaneously monitored in space two types of mRNAs, *ASH1* and *IST2* [19], and the other one performed *ARG3* mRNA tracking using a double-helix point spread function and included in-depth physical analysis of the different types of movement [6]. We now report three-dimensional tracking of mRNA movement in yeast undergoing profound structural changes associated with mating. In the present study, we compared *MFA2* mRNA surface tracking (2D) to spatial tracking (3D).

Our 3D tracking confirmed the occurrence of the three types of *MFA2* granule movement: vibrational, oscillatory and translocational (Figure 1), which were previously described in 2D tracks [27]. Only two types of movement: vibrational and oscillatory, were observed for the *PGK1* granules that distribute throughout a yeast cell (Figure 2). 3D tracking required collecting information from the entire cell volume and thus was necessarily performed at a relatively slow rate of image capture, 2–4 s/frame. The average velocity of translocating *MFA2* granules in 3D was estimated to be 0.15 µm/s (Figure 4) and was lower as compared to 2D analysis. This estimate is two times lower than the velocities determined for different mRNAs in budding yeast in 3D in studies for which the temporal resolution was greater [6,19]. The question then arose as to whether our estimate of 3D velocity reflected reality.

Currently, much greater temporal resolution is possible with 2D imaging, 0.55–0.07 s/frame, and sensitivity is greater, so small, ultra-fast granules of low fluorescence intensity can be tracked [27]. The average velocity of these translocating granules in 2D was 1.5 µm/s (Table 2), consistent with previous reports [24,27]. However, track displacement rates in 2D and 3D were similar, despite the coarseness of the 3D determination (Table 2). Therefore, the disparity between the average velocities of granules was mainly attributed to the difference in time resolution of the methods. Consistent with this interpretation, velocities for several granules tracked in 2D were recalculated for positions determined at 2 s intervals, and the resultant “intermediate” coarse grain values converged with the slower velocities determined for other cells by 3D tracking. Nevertheless, 3D tracking did reveal a substantial amount of movement in the z-direction, indicating that the rapid jumps observed in 2D tracking were not always as straight as they appeared. It remains to be seen whether faster velocities will be measured with even greater time resolution and since our 2D values ignored movement in the z-direction, the translocating mRNA granule velocity determined from our 2D approach must be considered to be a lower limit.

### 3.2. Complexity of MFA2 mRNA Transport

*MFA2* and *PGK1* mRNPs fluorescent labeling using a U1A plasmid-based system has shown differences not only in number and sizes of the granules for both RNP types, but also distinct dynamic behavior including motility properties (trajectory displacement, and length, Figure 1 and Figure 2, Table 1). However, we do not exclude the possibility that these differences were dependent on specific *cis*- signals located on the transcripts themselves and *trans*-acting factors associated with them. Therefore, RBPs incorporated in the mRNP complexes of both transcripts were probably distinct and may have conferred different properties.

The MSD parameter distinguishes intentional and directed movement from random movement [44]. A completely random walk (Brownian motion) is characterized by a zigzag trajectory of variable track displacement and a nearly linear MSD profile with a shallow slope. In our study, directed movement resulted in a parabolic MSD profile. Using this and other physical parameters in the present study, we demonstrated that *MFA2* mRNA translocational movement from the cell body to shmoo was extremely circuitous. Furthermore, transitions between directed transport and diffusive motion could be discerned. Statistical tests of the switching within a single trajectory between directed and confined modes of motion reveals that 21% are confined- restricted stationary movement, 4% are directed, and the remaining 75% are classifiable by default as consistent with diffusion [6]. In budding yeast, *ASH1* mRNAs reaching the bud neck suddenly jump directly to the bud tip [19]. We identified in mating yeast, many translocating granules undergoing a change in the type of movement (e.g., Figure 3 and Figure 6; Movies S3 and S5).

Changes in speed may occur after a change in granule composition. Sometimes we observed small granules coalescing to create larger complexes (see Movie S3, big central granule starting at the 10th second). Previously, actin patch assembly was observed in yeast that had formed a shmoo [45] and actin networks participate in mRNA localization as the shmoo grows [10]. Slight movement of an mRNA granule may occur due to oscillation of the actin fiber or result from tiny steps along an actin fiber that has some curvature [46]. In addition, assembly and disassembly of actin patches are very quick during the establishment and maintenance of cell polarity, with a patch lifetime of 11 ± 4 s [45]. Hence, granules might also pause at actin branching points, sites of actin remodeling, or in places where there are actin discontinuities. Furthermore, we never observed two granules following the same route to the shmoo, consistent with volatility in the “cell’s roads” and the existence of a wide network of roads.

The directed movement of *ASH1* mRNA and other bud localized mRNAs from the mother cell to the daughter cell uses the Myo4p, an actin-cytoskeleton-dependent motor protein [7]. In mating yeast, it was shown that actin cytoskeleton is also an essential cytoskeleton for polarized growth and secretion, though its role in mRNA transport was not confirmed in the past. Because the actin cytoskeleton is required for *MFA2* mRNA delivery (Figure 8), we can assume that when subgroup of the low intensity *MFA2* mRNA granule was moving in short steps, it was not carried by a motor protein on the actin [41], but during larger jumps (For example see Figure 3 and Figure 4, Movies S3 and S4), some motor protein may have participated in the process and supported the higher velocity of movement. The velocity of transit can be affected by the number of motor molecules that transfer the cargo [28]. Although *MFA2* mRNA localized in the shmoo, we failed to see mRNA granules being transported to shmoo in yeast lacking Myo4p. We could offer several explanations. First, as we previously mentioned, translocation is a relatively rare phenomenon, so in the absence of Myo4p, we just did not observe it. This possibility seems improbable given that we did observe translocation in WT yeast. Second, similar to the cortical ER inheritance mechanism that depends on the cytoskeleton but also relies on anchorage [47], it is possible that some mRNA moved through the cytoskeleton without motor proteins, but instead were associated with other organelles such as ER or secretory vesicles as a redundant mechanism and localized at the shmoo due to a strong affinity of the complex to that specific location. Transport and anchoring are distinct processes, for example, directed mRNA movement occurs from the cell body to bud and then granules switch to confined movement within the daughter cell [19]. Third, *MFA2* mRNA may have transported as individual transcripts, with fluorescence too weak for us to resolve [39], and aggregated at the shmoo as a visible granule. Finally, we see hints of a very fast transit mechanism involving oscillatory granules that underwent sudden, large displacements.

### 3.3. Which Motor Protein Participated in MFA2 mRNA Transport?

Class V myosins have biochemical and structural properties suitable for actin-based transport. In yeast, five genes of the myosin family are known to be expressed. Two are class V myosin; Myo2 and Myo4 are motor proteins that move along actin cables within the cell [48].

Myo4p protein participates in the transport of mRNA and ER in budding yeast [30,49] as well as in the localization of *SRO7* mRNA in mating yeast [14]. Furthermore, PB transport is dependent on the Myo4p/She2p RNA transport machinery in budding yeast [23]. We demonstrated that localization of large *MFA2* granules at the shmoo as well as cER inheritance persists without Myo4p (Figure S1). In the present study, granules moved at the same velocities in mutant yeast lacking Myo4p. *MFA2* mRNA did accumulate at the shmoo; however, directed movement of the granules from the cell body to the shmoo was never observed (Figure 9). Myo4p was identified as a principal motor of *ASH1* mRNA transport in budding yeast operating with a velocity of ~0.3 μm/s [7]. At first sight, this rate falls short of the measured *MFA2* mRNA granule velocity of ~1.5 µm/s for granules that undergo translocation in mating yeast, but those former calculations relied on videos acquired at 1 s intervals, which were 18 times slower that our acquisition rate in 2D. Therefore, Myo4p could mediate the fastest *MFA2* mRNA transport, but did not exist as the exclusive means of transport. Future study is required to figure out the alternative mechanisms of *MFA2* mRNA transport.

Myo2p is more typical for transporting vascular organelles, such as Golgi or peroxisomes, with velocities of movement of 0.15–0.45 µm/s [48]. It also mediates rapid, exocytotic-vesicle transport into the bud with a velocity of 3 µm/s [50]. Fungal RNA could be transported by vesicle in the case of extracellular vesicle-mediated export of nucleic acids [51], but *MFA2* mRNA granules are considered to be cytosolic bodies [27] that translate and undergo a series of biogenesis steps occurring in the cytosol [33,52]. Thus, we rule out the possibility that *MFA2* mRNA was packed into vesicles and then transported by Myo2p. Myo2p can associate with a large, RNP particle(s) and form a PB that is distinct from actively translating polysomes and exosomes [53], but confirmation that Myo2p provides a means for directed transport of mRNA will require additional study.

### 3.4. PB Involvement in MFA2 mRNA Transport

In mammalian cells, PB movement is not usually direct and is mediated by microtubules [54]. In budding yeast, the movement of PBs is undirected, is dependent on Myo4p/She2p RNA transport machinery and occurs along the actin cytoskeleton from the mother to the daughter cell. In cells that formed a shmoo, we showed 100% co-localization between Dcp2p and high intensity granules of *MFA2* mRNA and only 53% co-localization in low intensity granules. The formation of the *MFA2* mRNPs subtypes can be dependent on recently discovered properties of PBs. The formation of PBs depends on liquid-liquid phase separation (LLPS), which means that these non-membrane cellular compartments are formed by the phase separation from the cytoplasm. Moreover, several fluorescence microscopy studies have revealed that a rapid exchange of components (proteins and RNA) occurs between PBs and the cytoplasm, suggesting that PBs have dynamic and liquid-like properties that can affect not only their size, but also their properties [55,56,57].

We recorded the common transport of the PB component, Dcp2p, together with *MFA2* mRNA in yeast treated with pheromone α (Figure 7). The observation shows that in oscillatory and translocational modes, granules moved along with and without the PB protein, whereas in vibrational mode, granules all co-localized with Dcp2p. Dcp2 is one of the core-components of PBs; it contributes to PBs and contains a binding domain that is required for interaction with RNA molecules [58]. Based on our previous results and current observations, we characterized four subtypes of *MFA2* RNPs. One is *MFA2* mRNA/Dcp2p RNPs showing characteristics of classical PBs localized in the perinuclear region of the cell: high intensity and vibrational motile properties. These PBs are enriched in translationally repressed *MFA2* mRNA and are required for storage and sorting. The second subtype comprises medium intensity *MFA2* mRNA/Dcp2p RNPs granules located in the cytoplasm of the cell body with possibly modified protein composition and oscillation and translocation properties. This new PB type can be transported to the shmoo tip. It has been shown that concentrations of many PB-associated proteins with domains containing low complexity sequences and LLPS-associated RNA-binding activities play an essential role in the formation of new PBs. Regarding formation of new PBs, post-translational modifications of proteins, such as phosphorylation and ubiquitination, are known to have significant contributions [59,60]. The third subtype is low intensity *MFA2* RNPs that have the same translocational properties as the medium intensity one found in the cell body and shmoo. While it does not require Dcp2p for delivery, this subtype possibly has a distinct composition.

The finding suggested two scenarios that were not mutually exclusive. First, a fraction of fast *MFA2* granules translocated to the shmoo independent of PBs, whereas another pool moved as a PB complex. Second, an initial stage of the RNP complex formation excluded Dcp2p protein, but it joined later, just prior to movement toward and retention at the shmoo. During its life cycle, PB size is not constant; the PB can undergo several cycles of growth and decomposition [57,61,62].

There is a fourth subtype of MFA2 RNPs: Mating Bodies (MBs) located in the shmoo tip, which form from an accumulation of the second and third types of transported granules delivered from the cell body. The dynamics of these RNPs clearly indicate liquid-like properties such as spherical morphology and fusion of two PBs to form a larger structure that maintains its original round shape [62]. These granules are characterized by high intensity and vibrational motility like perinuclear *MFA2* mRNPs, which are formed from the accumulation of granules delivered from the nucleus. MBs and perinuclear granules have similar PB features characterized by high intensity, vibrational motile properties, and co-localization with Dcp2p, while they are distributed in different cell sub-compartments. The perinuclear granules are located in the cell body, while MBs are present in the shmoo tip. Moreover, they contain *MFA2* mRNA with a distinct status; perinuclear contain translationally repressed mRNAs, while MBs contain translationally capable mRNAs are required for translation of Mfa2p [27]. Hence, an RNP’s fate is tied to its complex and variable composition and different factors may control RNP movement at different stages of the journey and at their destination.

**Figure 10:**
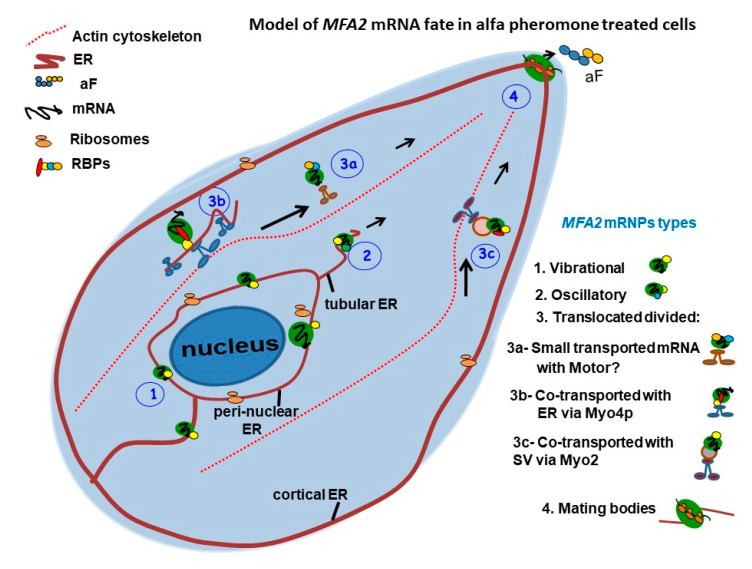
Model of *MFA2* mRNA fate in alfa pheromone-treated cells.

Based on our observations, we propose the following model of *MFA2* mRNA fate in shmoo forming cells (Figure 10). Cytoplasmic mRNAs are regulated at the post-transcriptional level and transcript localization is one important means for translation control. There are three subtypes of cytoplasmic *MFA2* mRNPs that determine cytoplasmic fate (vibrational, oscillatory and translocational), as well as a fourth RNP type called the *MFA2* MB located in the shmoo tip that is responsible for aF translation and subsequent secretion of aF from the cells. The *MFA2* RNPs are distinct in size, subcellular location, and numbers of RBPs associated with them, and therefore, they have different motility properties. First, immotile RNPs, located in the cell body, manifest as vibrational mRNA granules. From their properties and location, it seems likely that they sit on and attach to peri-nuclear ER. The second type of the RNPs moves quickly, although the movement is restricted to the cell body sub-region, therefore it comprises the oscillatory mRNP type. These RNPs are partially attached to some structure in the cell body, we assume that this type of granule can be attached also to tubular ER. These granules have dynamic structures that change in size and location during shmoo growth and are pulled to the bud/shmoo tip of the cell. [63,64]. *MFA2* RNP velocity can be dependent on the growth of tubular ER to the shmoo. Interestingly, tubular ER appears to exclude ribosomes, which means that translationally inhibited RNPs or PBs can be associated with it [63].

The third type of *MFA2* RNP is the translocated granule that moves to the shmoo. According to our results, some *MFA2* mRNA granules were transported to the shmoo via Myo4p though actin and showed complex movement. Since RNP delivery to the shmoo occurs in the absence of Myo4 motor, we propose that transport can be accompanied by tubular ER pooled to shmoo that then branches out to form new contacts in multiple directions with the PM to reestablish [47]. Therefore, tubular ER can be an alternative means for *MFA2* mRNA transport to the shmoo.

We also do not exclude the possibility that translocated mRNPs can be delivered to shmoo together with perinuclear-ER-derived secretory vesicles (SV). These vesicles accumulate early during shmoo growth [65]. These hypotheses should be investigated in future research.

## 4. Materials and Methods

### 4.1. Yeast Strains and Growth Conditions

The *S. cerevisiae* strains and plasmids used in this study are listed in Table 5 and Cells were grown at 25–30 °C in synthetic complete medium to the early exponential growth phase. For selection, the appropriate amino/nuclear acid was omitted from the media, since its expression was directed by the exogenous plasmid (see Table 6, yeast marker).

### 4.2. Labeling System/Plasmids

For in vivo mRNA labeling, two plasmids were transfected into yeast cells. One plasmid contained the chimeric U1A-GFP protein. The second plasmid included the target gene with a UA1-binding site (loops). Centromeric plasmid pSA03 contained an MFA2 promoter and its open reading frame, with a 3′-UTR that included 16 repeats of the UA1-binding site for in vivo fluorescent labeling of its mRNA molecules in living cells [66]. A full length MFA2 3′UTR fragment was amplified by PCR and inserted between SacI and Not1 site digest of pRP1193 using: forward primer, 5′-cggactagtccgccgcggcTTTTTGACGACAACCAAGAG where capital letters are the MFA2 3′UTR and small letters denote a SpeI restriction site and reverse primer, 5′-atttgcggccgcggcGCGGAGGGAAAGGCGTATC-3′, where capital letters are the MFA2 3′UTR and small letters denote a Not1 restriction site. The sequence was verified by Sanger sequencing. Centromeric plasmids carrying PGK1, whose 3′-UTR also contained 16 repeats of U1A-binding site (pPS2037), pU1A-GFP (pRP1187) and DCP2p-RFP (pRP1152), were kindly provided by Prof Roy Parker (Howard Hughes Medical Institute, University of Colorado Boulder, Boulder, CO, USA) (Table 6).

### 4.3. α-Factor Treatment

Stock solution of α-factor (Sigma-Aldrich, St. Louis, MO, USA) consisted of 1 mg/mL in methanol. Cells were grown in synthetic complete medium to early exponential phase and treated with 3 nM α-factor for 2 h. In some cases, the culture was collected by centrifugation and resuspended in a six-fold smaller volume just before the addition of α-factor. Only fresh cell samples of low concentration were inspected under the microscope (<15 min) to avoid adverse effects (e.g., starvation, hypoxia) of incubating them in between the slides.

### 4.4. Cytoskeleton-Disrupting Drug Treatment

The cells expressed fluorescent labeling *MFA2* mRNAs were treated with αF for 1 h and were treated with Benomyl (Chem Service, West Chester, PA, USA PS-222, stock concentration 10 mg/mL in DMSO) in final concentration 40 μg/mL or with Latrunculin A (Tocris, Bristol, UK, 3737, stock concentration 9.1 mg/mL in DMSO) in final concentration 84 μg/mL during 150 min at 30 °C, 150 rpm. The cells were fixed with 4% Formaldehyde (Sigma, St. Louis, MO, USA) after 30, 60, 90, 120 and 150 min after the treatment and examined by fluorescence microscopy.

### 4.5. Image Processing and Analysis of Time Series Movies

The 3D and long time-lapse videos of mRNA granules and proteins in mating yeast were taken at room temperature (∼25 °C) by confocal microscope using a 63×/1.40 NA objective (LSM 700; Carl Zeiss, Jena, Germany). In these experiments, cells were typically imaged in 3D (six–seven z-planes per time point), at 0.5 µm steps. The time point duration 2–4 s 2D recording with two fluorescence filters was done with a fluorescence microscope (Olympus IX81, Objective: 100× PlanApo, 1.42 numerical aperture). The following wavelengths were used: for GFP, excitation at 480 nm and emission at 530 nm; for RFP, excitation at 545 nm and emission at 560–580 nm. There was a 2.5 s delay between the green and red fluorescence measurements. For imaging two fluorophores in a cell, sequential screening was performed to avoid overlapping with the LSM image. The AxioVision LE version 4.8.2.0 Zeiss, Jena, Germany software was used for producing 3D movies. Labeled MFA2 and *PGK1* mRNA were tracked and the movies presented as a z-series compilation of 7–10 photographs in a stack (0.5 µm) using the AxioVision LE version 4.8.2.0, Zeiss, Jena, Germany software. The quantitative co-transport analysis of the *MFA2* mRNA/Dcp2p-RFP over a period of time was carried out in Imaris 7.4.2 software (Bitplane AG, Zurich, Switzerland).

The short videos of *MFA2* and *PGK1* granules were produced at room temperature (∼25 °C), using the inverted motorized fluorescent microscope with a 100×/1.30 objective (Axio Observer.Z1; Carl Zeiss, Jena, Germany), an LED light source (Colibri) and a high speed camera (Zeiss HS; Carl Zeiss, Jena, Germany). Videos were made using AxioVision Rel. 4.8 imaging program (Carl Zeiss, Jena, Germany). The camera had a peak QE of more than 90%, making it a near-perfect detector that could capture images at high speed with an unequaled signal-to-noise. This high sensitivity allowed us to follow the low intensity, faster, transported granules in the cell.

For presentation of the movies, the 3D image sequences were transformed into a time sequence using the maximum projection option to 3D. Videos were processed using Imaris 7.4.2 software (Bitplane AG). Statistical data analysis was performed using Excel 2010 version 14 (Microsoft, Redmond, Washington, USA). The final images were prepared with Photoshop CS6 version 13.0.06 (Adobe, San Jose, CA, USA).

### 4.6. Tracking Analysis of MFA2 or PGK1 Granules

The 2D and 3D trajectories of *MFA2* and *PGK1* granules that were acquired using a confocal microscope (Fast Acquisition, LSM 700; Carl Zeiss, Jena, Germany) at room temperature (∼25 °C) were built using the semi-manual mode of the Imaris 7.4.2 program (Bitplane AG, Zurich, Switzerland). The granules chosen for analysis had at least 40 visible time points without unviable gaps exceeding three consecutive time points. 3D movies had several z-stacks in which only one or two of them caught the granule. For analysis, we superimposed all z-stacks at each time point, then drew a straight line between time points. This meant that there were epochs when the granule movement could not be determined accurately and the track was assumed to be smooth, but was in reality, most likely to be kinked.

Local or instantaneous velocity was calculated using an interval of three time points. The displacement, duration, track length, and types of tracking were analyzed according to intensity and position of the mRNA granules in the cell using the same program. Mean velocity was calculated as the total track length divided by the observation period. Except where specifically stated otherwise, track displacement and track length were given for a standardized track duration of 60 s. In cases for which track durations greater than 60 s were available, values were determined for 60 ± 5 s segments and then averaged. Vantage images show the track pathway in 2D/3D scale using Imaris 7.4.2 program (Bitplane, Zurich, Switzerland).

MSD, log(ψ), and angle of *MFA2* and *PGK1* granular motion were analyzed based on x, y, z, and time parameters, obtained from track building. MSD profiles were calculated for each trajectory by a classical Brownian motion algorithm [42], and plotted as a function of τ. τ is the lag time between the two positions taken by the particle. For log(ψ) analysis, the route was divided into sub-tracks of 100-time frames (t_w_)_;_ in each sub-track the maximum displacement value was defined (R). The diffusion coefficient (D) was calculated from the derivative of the MSD function. Then probability, log(ψ) was calculated by taking the linear approximation of Dt_w_/R^2^ which will be valid for R < sqrt(Dt).

Log(ψ) = −0.125–0.25(Dt_w_/R^2^) (1)

The measured probability was compared to a set threshold (ψ = 0.5). A probability ψ > 0.5 would indicate an increased particle distance within a finite time, having lost the ability to be confined [67]. More details on the fitting algorithm can be found in Supplementary Materials.

## Figures and Tables

**Table 1:** Range of motion parameters for *MFA2* and *PGK1* granules tracked in 3D.

Type of Motility	Vibrational		Oscillatory		Translocational	
Type of mRNA	*MFA2*	*PGK1*	*MFA2*	*PGK1*	*MFA2*	*PGK1*
Track length (µm)	2.5 ± 1.4	5.5 ± 2.6	6.5 ± 3.1	14.6 ± 3.9	6.4 ± 2.3	---
Track displacement (µm)	0.5 ± 0.2	0.6 ± 0.2	1.1 ± 0.3	1.5 ± 0.4	2.3 ± 0.7	---

A total of 150 granules were analyzed in 47 cells for 60 s periods. Track lengths and track displacements are given as mean ± SEM.

**Table 2:** 2D vs. 3D comparison of translocating *MFA2* granules.

Tracking Mode	Frame Period (s)	Track Displacement Rate (μm/s)	Mean Velocity (μm/s)
2D	0.055–0.070	0.04 ± 0.01	1.5 ± 0.7
3D	2–4	0.03 ± 0.01	0.15 ± 0.06

10 granules were analyzed for each tracking mode. Mean velocity was calculated as the total track length divided by the observation period and track displacement rate as the track displacement divided by the observation period. Observation periods varied from 15 to 250 s. Values are given as mean ± SEM.

**Table 3:** Velocities of MFA2 granules with and without PB protein.

Type of Motility	*MFA2* mRNA with Dcp2p	Dcp2p	*MFA2* mRNA
Vibrational	0.05 ± 0.04	0.05 ± 0.04	---
Oscillatory	0.15 ± 0.01	0.15 ± 0.03	0.22 ± 0.07
Translocational	0.13 ± 0.03	0.13 ± 0.03	0.23 ± 0.01

Mean velocity = track length/track duration, in units of µm/s. 85 granules, 0.2 to 0.8 µm in diameter, were analyzed in 16 cells. PB granules were defined by the presence of Dcp2p (see text). Data are given as mean ± SEM.

**Table 4:** Motility types of *MFA2* granules in *Δmyo4* vs. WT strains.

Type of Motility	Velocity in WT (µm/s)	Velocity in *ΔMyo4 (*µm/s)
Vibrational	0.08 ± 0.07	0.08 ± 0.06
Oscillatory	1.0 ± 0.2	1.1 ± 0.3
Oscillatory with large displacement	1.7 ± 0.3	1.6 ± 0.4
Translocational	1.5 ± 0.4	----

Mean velocity = track length/track duration, in units of µm/s. 100 granules, 0.2–0.8 µm in diameter, were analyzed in 20 cells. Data are given as mean ± SEM.

**Table 5:** *S. cerevisiae* strains used in the study.

Strains	Genotype	Source
ySA061	*MATa, ura3, his3, leu2* [pPS2037, pRP1187]	Aronov et al., 2015
ySA056	*MATa, ura3, his3, leu2* [pSA03, pRP1187]	This study
ySA022	*MATa, ura3, his3, leu2* [pSA03, pRP1187, pRP1152]	This study
ySA118	*MATa, ura3, his3, leu2, myo4: NEO;* [pSA03, pRP1187]	This study
ySA180	*MATa, ura3, his3, leu2,* [pSA05, pRP1194]	This study

**Table 6:** Plasmids used in the study.

Plasmid ^a^	Genotype	Yeast Marker	Source
pRP1187 ^b^	*GPDp-U1A*-GFP	LEU2	P. Silver ^c^; Brodsky and Silver, 2002
pMC313	*GPDp-MFA2-U1Ax16-3′UTR of MFA2*	URA3	Aronov et al., 2015
pPS2037	*PGK1p-PGK1*-*U1Ax16*-*3*′*UTR of PGK1*	URA3	S. Aronov ^d^; Aronov et al., 2015
pSA03	*MFA2p-MFA2*-*U1Ax16-3*′*UTR of MFA2*	URA3	This study
pSA05	*MFA2p-U1Ax16*	URA	This study
pRP1152	*DCP2p-DCP2*-RFP	HIS3	R. Parker ^e^

^a^ All plasmids, unless otherwise indicated, contained CEN and therefore were present in one or two copies per cell. ^b^ Multicopy 2 μ plasmid. ^c^ Harvard Medical School, Boston, MA, USA. ^d^ Ariel University, Ariel, Israel. ^e^ Arizona, Tucson, USA.

## Supplementary Materials

The following are available online at https://www.mdpi.com/2073-4409/9/10/2151/s1, Figure S1: MFA2 mRNA distribution and cortical ER inheritance is not dependent on Myo4p motor protein, Appendix S1, Movies S1–S7.
